# Evaluating Alternatives to Locomotion Scoring for Detecting Lameness in Pasture-Based Dairy Cattle in New Zealand: In-Parlour Scoring

**DOI:** 10.3390/ani12060703

**Published:** 2022-03-11

**Authors:** Chacha W. Werema, Dan A. Yang, Linda J. Laven, Kristina R. Mueller, Richard A. Laven

**Affiliations:** 1 School of Veterinary Science, Massey University, Private Bag 11 222, Palmerston North 4442, New Zealand; l.j.laven@massey.ac.nz (L.J.L.); k.mueller@massey.ac.nz (K.R.M.); r.laven@massey.ac.nz (R.A.L.); 2 College of Veterinary Medicine and Biomedical Sciences, Sokoine University of Agriculture, Morogoro 67115, Tanzania; 3 College of Veterinary Medicine, Nanjing Agricultural University, Nanjing 210095, China; d.yang@massey.ac.nz

**Keywords:** lameness, locomotion scoring, in-parlour scoring, decision tree, machine learning, pasture-based system, dairy cows

## Abstract

**Simple Summary:**

Lameness in dairy cows is a significant challenge globally. Early detection accompanied by effective treatment can reduce the number of cows that are lame and the impact of lameness. Currently, locomotion scoring by observing the gait posture of cows is the most widely used method of detecting lame cows. However, its use is limited, especially in pasture-based production systems like in New Zealand. One possible alternative to locomotion scoring is observing and recording cows for indicators of lameness while cows are being milked. We recorded the presence of four indicators (shifting weight, abnormal weight distribution, swollen heel or hock joint, and overgrown hoof) on two dairy farms in New Zealand. Two or more indicators were more useful predictors of higher locomotion scores (lameness). However, more results on more farms are needed before the in-parlour scoring procedure can be recommended as an alternative to locomotion scoring in pasture-based dairy cattle.

**Abstract:**

Earlier detection followed by efficient treatment can reduce the impact of lameness. Currently, locomotion scoring (LS) is the most widely used method of early detection but has significant limitations in pasture-based cattle and is not commonly used routinely in New Zealand. Scoring in the milking parlour may be more achievable, so this study compared an in-parlour scoring (IPS) technique with LS in pasture-based dairy cows. For nine months on two dairy farms, whole herd LS (4-point 0–3 scale) was followed 24 h later by IPS, with cows being milked. Observed for shifting weight, abnormal weight distribution, swollen heel or hock joint, and overgrown hoof. Every third cow was scored. Sensitivity and specificity of individual IPS indicators and one or more, two or more or three positive indicators for detecting cows with locomotion scores ≥ 2 were calculated. Using a threshold of two or more positive indicators were optimal (sensitivity > 92% and specificity > 98%). Utilising the IPS indicators, a decision tree machine learning procedure classified cows with locomotion score class ≥2 with a true positive rate of 75% and a false positive rate of 0.2%. IPS has the potential to be an alternative to LS on pasture-based dairy farms.

## 1. Introduction

Early lameness detection is one of the most significant challenges in the dairy industry. The impact of delayed lameness detection and treatment is evident in terms of production losses [1,2,3], treatment costs [4], fertility problems [5,6,7], and health and welfare issues [8,9,10], as well as chronic irreparable claw damage [11].

Earlier detection accompanied by effective treatment would reduce the prevalence and impact of lameness [12]. However, early diagnosis requires effective detection methods that can be easily employed on the farm. Currently, locomotion scoring (LS) is the most commonly used method of lameness detection on-farm [13]. However, in pasture-based production systems such as those which predominate in New Zealand, opportunities for LS are generally limited to around milking time, with, ideally, the cows being observed as they exit the milking parlour after milking. This practice requires trained staff (in addition to those needed for milking) to stand outside the parlour exit for the whole of the milking session. However, in New Zealand, dairy farms are generally large with a high ratio of cows to staff (mean 146 cows per full-time equivalent staff member [14]). Thus, ensuring staff availability for LS during a period of high workload can be challenging to achieve. Additionally, especially on farms with herringbone parlours, it can be challenging to individually observe every cow when they leave the parlour because cows exit the parlour in batches. This difficulty is exacerbated by environmental factors, such as wind, sunlight, and rainfall, that the observer and cows might be exposed to during the scoring exercise. Consequently, there is a need for easy to use and effective lameness detection systems in pasture-based herds.

An alternative to visual LS would be to observe cows for indicators associated with lameness during milking. This practice could allow a staff member to milk and record lameness simultaneously, especially if the aim were to determine lameness prevalence in a herd (which requires scoring only part of the herd [15]) rather than individually identifying lame cows.

The critical problem with detecting lameness in cows during milking is that it is impossible to observe gait, which is crucial for most locomotion scores. However, the same problem arises when detecting lameness in cows in tie stalls. Leach et al. [16] developed a stall lameness score (SLS) protocol to detect lameness in tied cows using indicators/behaviours such as weight shifting, rotation of feet, standing on the edge of a step, resting of feet, and uneven weight-bearing. They compared lameness based on SLS, where a cow was defined as lame by the presence of two or more indicators on the screening list, with a 5-point scale [10] gait-based locomotion score. The study found that the SLS underestimated the proportion of lame cows compared to LS.

The study by Leach et al. [16] was a small one (including only 98 cows) and was thus more a proof of concept than a definitive test of the SLS value as a lameness measure. However, multiple studies have since used the SLS (or a modification) to assess lameness in dairy cows in tie stalls [17,18,19,20,21,22]. For example, both Gibbons et al. [22] and Palacio et al. [21] compared SLS using the indicators described by Leach et al. [16] except for foot rotation, with LS systems [23,24]. Both used the presence of two or more predictors to define a lame cow using SLS. However, while Palacio et al. [21] concluded that using SLS resulted in fewer cows recorded as lame, Gibbons et al. [22] reported that SLS overestimated lameness prevalence compared to LS. Nevertheless, both studies concluded that the SLS was a helpful measure of determining lameness prevalence at the herd level.

Another opportunity where standing cattle could be observed for lameness is when they are locked in stanchions. For example, Hoffman et al. [25] observed cows locked in stanchions for the presence/absence of an arched back, cow-hocked stance, widely placed hind limbs, and favouring a leg while standing. These individual indicators were compared with LS (Sprecher et al. [7]), with a locomotion score ≥ 3 on a 5-point scale used to define lameness. Hoffman et al. [25] concluded that observation of cattle locked in stanchions lacked sufficient sensitivity or specificity to be used as an alternative to LS. However, they suggested that observing these indicators may be useful as a screening test for identifying cows requiring a further examination for lameness.

García-Muñoz et al. [26] also investigated the association between postural and gait abnormalities observed in cows locked in stanchions utilising the same indicators and lameness definition as [25]. Consistent with Hoffman et al. [25], they concluded that the sensitivity of lameness detection in stanchions was too low for it to be used as an alternative to standard LS.

These studies suggest that non-locomotory lameness assessments may be a suitable method for identifying cows that need to be more closely examined for lameness or for estimating lameness prevalence in a herd. However, none of these studies has been undertaken in pasture-based dairy cattle during milking in rotary parlours. The latter is important, as rotary parlours tend to be used in larger herds where the number of cows per full-time staff member is higher [27], increasing the value of alternatives to standard LS. Nevertheless, not all indicators proposed by Leach et al. [16] can be assessed effectively in cows milked in rotary parlours, particularly evaluating uneven weight-bearing while moving the cow from side to side or standing on the edge of a stall. Thus, we need additional new indicators that can be assessed by observation only for in-parlour scoring. Potential indicators include back-arching [25,26], overgrown hooves [28,29,30], claw injuries [31,32], swelling of hock or heel [33,34] and swelling around the coronary band [35]. These indicators could be used alongside abnormal weight distribution and shifting weight to score the risk of lameness in the milking parlour. Therefore, this study aimed to assess the feasibility of observing these indicators during milking and compare this in-parlour scoring (IPS) procedure with whole herd LS in pasture-based dairy cows.

## 2. Materials and Methods

### 2.1. Animals and Farm Location

This study was conducted in two dairy farms located in the Manawatu region on the North Island of New Zealand. Both farmers were clients of the Massey University Farm Practice, and they were interested in participating in this project. Both farms used a rotary milking parlour and milked cows twice daily. On both farms, animals were kept at pasture permanently, and cows were given a small amount (~1 to 2 kg/cow) of additional feed at milking time. Hoof trimming and LS were not routine management practices on either farm. However, as part of another study, 250 cows were assessed and trimmed as required by a professional hoof trimmer on two occasions while the current study was being undertaken on farm 1. Lame cows were identified by farm staff when they were brought in for milking.

Farm 1: This farm had 1200 dairy cows available for study, with spring and autumn calving groups. Most cows were Friesian and Jersey crossbreds, with approximately 10% Friesian cows. Cows’ age ranged from 2 to 10 years, with a mean age of 4 years. The milking herd was managed as two groups and milked twice daily through a 60-unit rotary milking parlour. Each group was milked in succession and grazed on separate paddock rotations within the same farm. On this farm, routine lame cow management involved regular veterinary visits every two weeks to treat lame cows, maintaining a lame-cow group kept close to the milking parlour and milked once a day in the morning. The lame-cow group was not included in this study as all farm visits were in the afternoons. According to on-farm treatment records of 50 lameness cases throughout the lactation season, the leading causes of lameness were white line disease (54%), sole injury (16%) and foot rot (8%). No digital dermatitis was diagnosed at any time.

Farm 2: This farm had 400 dairy cows calving in spring; approximately 95% were Jersey cows, with Friesian and crossbreds accounting for 5%, with an average age of six years. This farm used a 44-unit rotary milking parlour, managed the milking herd in two groups, and grazed in separate rotations on the same farm. However, there was no independent lame group (all lactating cows were milked twice daily). Lame cows were routinely treated by the farmer, with cows receiving veterinary services on request. According to on-farm treatment reports of 29 lameness cases across the lactation season, the leading cause of lameness was white line disease (72.4%). No digital dermatitis was detected at any time during the study.

### 2.2. Locomotion Scoring

Prior to the study commencing in August 2018, the first author (a veterinarian) was trained in LS. The training consisted of observing training videos created by DairyNZ [36] and Agriculture and Horticulture Development Board (AHDB) [37], followed by supervised LS on-farm (live cows) with a trained and experienced observer until the trainer was satisfied that the trainee could perform LS effectively. Inter-observer agreement between trainer and trainee was substantial (kappa = 0.870; 95% CI: 0.771–0.926). Cows were scored as they left the parlour after milking. The LS evaluation area was a flat concrete surface about 20 m in length, a walking distance sufficient for the assessment of animals’ gait and posture attributes while they were exiting the milking parlour.

Locomotion was scored by the first author using the DairyNZ lameness score. This scoring system has been adapted from the Agriculture and Horticulture Development Board AHDB mobility score to create a system that can be used to score cattle when they are walking back to pasture after being milked [38]. The DairyNZ lameness score is based on the co-assessment of walking speed, walking rhythm, weight-bearing, back alignment, head position, stride length, and foot placement on a 4-point scale from 0 to 3 (Table 1).

Data were collected monthly on both farms, with whole herd LS being carried out a day before the in-parlour scoring procedure. This study was undertaken from August 2018 (start of lactation) until the end of that lactation season (April 2019). Farmers received feedback regarding cows identified as lame by LS.

### 2.3. In-Parlour Scoring (IPS)

Due to the rotary platform’s high speed (10 min for one rotation) during milking, it was not possible to score every individual cow. Consequently, every third cow’s hind limbs were observed (at a distance of ~1 metre) during afternoon milking and visually screened for the presence or absence of the prepared checklist of indicators, summarised in Table 2.

### 2.4. Statistical Data Analyses

Initially, all data were processed using an Excel spreadsheet (Microsoft, Seattle, WA, USA). Then, data were put forward for analysis only from cows with a locomotion score followed the next day by an in-parlour score. We used SPSS version 25 (IBM Corporation, Armonk, NY, USA) for all analyses except where stated otherwise. Descriptive statistics were created for each dataset. The correlation between the presence/absence of an IPS indicator and the other IPS indicators and the presence/absence of locomotion scores ≥ 2 was assessed using the Phi correlation coefficient to ensure there was no collinearity. Then for each individual IPS indicator and the presence of at least one, at least two, and at least three positive IPS indicators, the sensitivity, specificity, positive and negative predictive values for predicting locomotion scores ≥ 2 were calculated (MedCalc Version 19.5.1; MedCalc Software, Ostend, Belgium).

The ability of IPS to predict actual locomotion score (0, 1 or ≥2) was then analysed using a decision tree (DT) machine learning method. This was implemented using Scikit-learn—a machine learning library for the Python programming language [40]. The DT method was used to classify a cow observation into a locomotion score class based on the input of the IPS indicators. For this analysis, the amount of information that a specific IPS indicator conveyed was measured using Gini impurity. During the training process, splits were chosen by maximising the decrease in the Gini impurity (which is calculated by subtracting the weighted impurities of the branches from the original impurity). If a node (decision point) is entirely pure, i.e., observations are all classified into one class of locomotion score, then Gini impurity equals 0, no further splits will be performed. For this training process, the data were randomly split into four folds, with each fold containing approximately 25% of the entire observations.

The locomotion scores in one fold were kept as close as possible to the other folds, approximating the distribution of the locomotion scores estimated based on the entire observation set. One fold was held out as a test set, while the remaining three were used as a training set. This procedure was repeated four times, with each of the folds used once as test data (4-fold cross-validation). For each pair of training and test datasets, one DT was firstly grown to its maximum depth, i.e., no more splits at nodes were available (leaf nodes were pure or all IPS indicators had been used in a branch). The DT was then pruned based on the following criteria: (1) >20 observations were required to split an internal node, and (2) a split at a node had to decrease Gini impurity by at least 0.005. The value of 0.005 was chosen to balance the classification accuracy and the complexity of a DT. A shallow DT can lead to inaccurate classification, while a deep DT can give unreliable classification (i.e., the number of observations in a node is small or a split results in two nodes where the decision is made based on the one with a slightly larger number of observations).

The known locomotion score (recorded by the first author) and predicted locomotion scores from each DT classifier were then identified and organised into confusion matrices which were used to calculate the test accuracy for each DT classifier. The test accuracy is the proportion of all the observations in the test data that are correctly classified (i.e., the ratio of true positives and true negatives to the total number of observations). The DT classifier with the highest test accuracy was chosen for further interpretation and visualisation, and the true and false positive rates and precision were calculated for that DT classifier. True positive rate is the proportion of observations correctly classified into a specific class (i.e., ratio of true positives to the total of true positives and false negatives). It is equivalent to sensitivity (although it is important to notice that this is the sensitivity of a classifier, not of an individual IPS scoring method). False positive rate is the proportion of observations that were incorrectly classified into a particular class (i.e., ratio of false positives to the total of false positives plus true negatives). It is equivalent to 1—specificity (of a classifier, not of an individual IPS scoring method). Precision is the proportion of observations identified as belonging to a class that were correctly classified into that class (i.e., ratio of true positives to the total of true and false positives).

## 3. Results

### 3.1. Locomotion Score Distribution

The distribution of locomotion scores (which had matching in-parlour scores) for each visit to both farms is presented in Table 3. As score 3 cows were scarce (<0.3% of all scores), data from score 2 and score 3 cows were amalgamated (locomotion scores ≥ 2/lame cows). The data for each in-parlour scoring indicator on both farms are summarised in Table 4.

Of the seven potential indicators identified in Table 2, three were not found to be useful in the present study analysis because of the following: Firstly, only one cow was observed with a bruise/cut on the claw during the entire study period, so this indicator was excluded from the analysis. Additionally, swelling around the coronary band was not easily observed in either parlour due to poor light conditions and dirty feet. Finally, back arching was not easily detected as the milking platforms were too high compared to the observer’s eye line. This left four scoring indicators: shifting weight (SW), abnormal weight distribution (AWD), swollen heel or hock joint (SHH) and overgrown hoof (OH).

### 3.2. Assessment of the Association between the In-Parlour Scoring Indicators and Locomotion Score

Phi coefficients for the association between the presence/absence of the IPS indicators and LS are presented in Table 5. The strength of the association between the four IPS ranged from negligible to weak [41]. In contrast, the Phi coefficients indicated that the association between the in-parlour scoring indicators and locomotion score ≥2 was relatively strong for all four indicators [41] (Table 5).

### 3.3. Sensitivities, Specificities, and Other Test Measures

The sensitivity and specificity for predicting LS ≥ 2 of the four individual indicators and thresholds of 1, 2, and 3 indicators are shown in Table 6. Sensitivity and specificity data separated by the farm are presented in Appendix A Table A1. Using the presence of two IPS indicators to predict LS ≥ 2 maximised specificity and sensitivity (>98% and >93%, respectively, Table 6).

### 3.4. Association of In-Parlour Scoring Indicators and Locomotion Scoring (Decision Tree Method)

**Figure 1:**
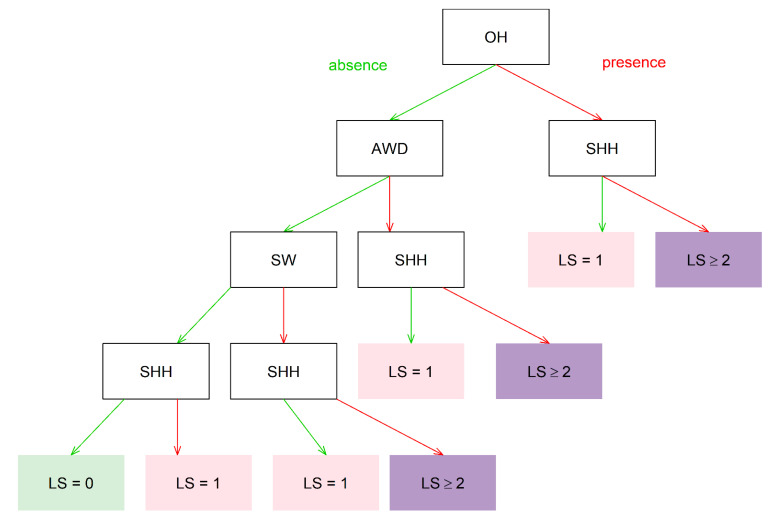
A decision tree to classify cows into different locomotion scores using observed in-parlour scoring indicators (*n* = 3095). *n*: the number of observations in the training set. Illustration: The green arrow from the root node pointed to the internal node when the indicator was absent. In contrast, the red arrow pointed to the internal node when the indicator was present. LS = locomotion score, SW = shifting weight, AWD = abnormal weight distribution, SHH = swollen heel or hock joint, OH = overgrown hoof.

The DT was used to classify cows into different locomotion scores based on observed IPS indicators. The DT with the highest accuracy is visualised in Figure 1. This classifier correctly classified 995/1030 (96.6%) of cow observations into locomotion score class recorded by the first author. For each locomotion score, the number of cow observations correctly and incorrectly classified are summarised in Table 7. For example, no lame or severely lame cow (locomotion scores ≥2) was classified as sound by the DT, and similarly, no cow with a locomotion score of 0 was classified as lame or severely lame with the DT.

True positive rate (TPR) and false positive rate (FPR) were lowest for locomotion score ≥2 and highest for locomotion score 1. In contrast, precision was lowest for locomotion score 1 and highest for locomotion score 0 (summarised in Table 8).

## 4. Discussion

The present study aimed to evaluate the potential of the in-parlour scoring (IPS) technique for detecting lameness in pasture-based dairy farms compared to visual LS. This is a preliminary study with a single observer on only two dairy farms, so further research is required. However, starting from seven indicators, we identified five indicators that were measurable while cows were being milked; four of these were shown in the subsequent analysis to be useful predictors of locomotion score.

The proportion of lame cows seen in the present study was consistent with previous reports of lameness in New Zealand. Lameness prevalence (percentage of locomotion scores ≥ 2) was 3.1 and 3.6% for farms 1 and 2, respectively (Table 3). These results are consistent with the range of prevalence reported by Fabian et al. [38], with the caveat that the true prevalence of high locomotion scores would have been greater on Farm 1 as no cows in the lame group were scored. However, the number of lame cows in the lame cow group was always <10 during the study. In addition, the pattern of lameness over a lactation season on both farms was similar to that reported by Lawrence et al. [42], who reported that the peak clinical lameness occurred during winter and the late spring for autumn-calving and spring-calving cows, respectively.

### 4.1. Feasibility of IPS

The present study’s main challenge was the rotary milking platform’s high speed (10 min for one rotation). As a result, there was insufficient time to screen all milking cows using IPS; however, it was simple to record the identity of all screened cows. In contrast, it was simple to locomotion score most (though not usually all) cows walking back to pasture after milking in a rotary parlour, but accurately identifying scored cattle was difficult. In fact, if identification is required (e.g., if the scoring is being used to identify cows for treatment), the proportion of cows that can be scored during a single milking is significantly reduced with LS compared to IPS. Thus, although the IPS technique takes more time per cow than LS, the ease of identification, combined with IPS not needing additional staff during milking, may mean that extra time per identified lame cow is similar for IPS and LS. However, further investigation on more herds, including farms with herringbone parlours (where in-parlour identification may be more difficult), is required to test this hypothesis.

Of the seven potential indicators included in the IPS at the start of this study, three indicators were not progressed to the analysis. Only one cow was observed with a bruise/cut on the claw during the entire study period; too few observations for inclusion in the analysis. Further research on more farms is required to identify whether this low level of claw injury is typical of New Zealand dairy farms. If it is, then observed claw injury would be unsuitable for in-parlour lameness scoring, although an observed claw injury indicator may be useful to record when cases are seen. Parlour design meant that arching of the back could not be observed on either farm as the observer had to stand below the level of the cows. On some rotary parlours, an observer can stand at the level of cows, and in herringbone parlours, the elevation of the cow may not be such an issue. So further investigation of the back arch as an in-parlour indicator of lameness in pasture-based dairy cattle is warranted. In addition, poor light conditions and dirty feet limited the observation of coronary band swelling. Therefore, it would be necessary to use a technique similar to that used by Yang et al. [43] to detect digital dermatitis lesions, i.e., wearing a head torch and washing the feet of all cows before scoring, to check effectively for coronary band swelling. Using this technique would increase the detection of digital dermatitis lesions but would undoubtedly increase the time taken for the IPS procedure. Therefore, further research is required to establish whether including observation of coronary band swelling improves IPS as an alternative to LS in herds where digital dermatitis is expected, as in New Zealand, is currently an extremely rare cause of lameness [43].

### 4.2. Assessment of IPS as a Method of Detecting Lame Cows

The four IPS indicators used in the analysis were independent of each other (phi < 0.13); thus, they provide different sources of information and, therefore, can usefully be used together as predictors of locomotion score. Individual indicators all had poor sensitivity for detecting locomotion score ≥ 2 (<50%), except for SHH, which had a moderate sensitivity of 77%. In contrast, specificity was high >90% for all indicators except OH, with a specificity of 85% (see Table 6). These findings are consistent with the conclusions of previous studies undertaken in tie-stall production systems that one indicator alone was not suitable for lameness detection [16,21,22]. Thus, as in those previous studies, we combined indicator scores to optimise lameness detection. Our analysis showed that using at least two positive IPS indicators was optimal (maximising sensitivity plus specificity). This result is consistent with previous studies [21,22] that also found that the presence of two or more indicators was optimal for identifying lameness in cows in tie stalls. However, our specificity and, in particular, sensitivity were better than reported in those studies, with Gibbons et al. [22] reporting a sensitivity of 63% and a specificity of 77%, and Palacio et al. [21] a sensitivity of 59% and a specificity of 90%, whereas we found a specificity of 98% and a sensitivity of 93%. In contrast, Leach et al. [16] concluded that optimal accuracy was obtained either when at least two of their indicators were positive or when any one indicator was present (excluding foot rotation). However, they reported specificity of ≥93% and sensitivity of ≤68% using at least two indicators to determine lame cows.

Our higher specificity and sensitivity compared to stall lameness scoring in tie stalls [16,21,22] may, in part, be related to the present study being undertaken in cows during milking without any physical contact, whereas stall lameness scoring involves physical contact to push the cow from one side to another. Physical handling produces stress which may reduce observed pain-related behaviours [44]. However, it is also likely that differences in our indicators could be responsible as of our four indicators; two (SHH and OH) were not used by previous studies [16,21,22]. Nevertheless, this study was performed on only two farms, so further research on more farms is required to better establish the sensitivity and specificity of IPS as a method for detecting locomotion scores ≥ 2.

In addition to analysing the ability of IPS to discriminate between lame and non-lame cows, we used a simple machine learning process to estimate how effective IPS was at classifying whether a cow had a locomotion score of 0, 1 or ≥2. As this process maximised accuracy across all three classifications, the results are different from the conventional analysis, which maximised accuracy for separating cows with a locomotion score of ≥2 from cows with other locomotion scores. Nevertheless, for locomotion scores 0 and 1, we obtained high sensitivity (or TPR) and high specificity (1-FPR). For locomotion score ≥ 2, specificity was extremely high (99.8%), but sensitivity was only moderate (75%). The difference between the two analyses is that the conventional analysis classified a cow with a locomotion score of ≥2 based on any two indicators; the decision tree classified any cow where SHH was absent as having a maximum locomotion score of 1 (see Figure 1).

As in our previous study with infrared thermography [45] and other studies which have evaluated similar scoring systems [16,21,22], we have used LS as a ‘benchmark’ to define lameness, although it does not have 100% specificity or sensitivity [23,24,46]. Thus, the differences between LS and IPS (or stall lameness scoring (SLS)) could be due to LS (even when correctly recorded) incorrectly categorising lame cows rather than errors in the other systems. In the present study, lameness prevalence based on two or more IPS indicators was higher than that recorded using LS (4.6% vs. 3.4%, respectively). However, this result was consistent over both farms (Appendix A Table A1). If these apparent false positives reflect cows that will become lame, using IPS might allow earlier lameness detection (and thus more effective treatment), especially if it can be done more frequently than LS. Previous studies of SLS and LS have been inconsistent, with some studies identifying more cows as lame using ≥2 indicators of SLS compared to LS [22], and others fewer [16,21]. Thus, the suggestion that IPS could be more sensitive than LS needs testing on more farms. Such research would also need to investigate the association between IPS and hoof lesions, especially in cows with a locomotion score of 1 and two IPS indicators.

This future research would also be an opportunity to address the findings of the DT process, in particular, to confirm that the DT presented in Figure 1 is the optimal tree and to identify whether combining multiple results from multiple IPS events would improve sensitivity. In addition, the value of combining such results in a machine learning process with other indicators, such as behaviour, milk production and live weight, that have been associated with lameness [47] should be evaluated.

## 5. Conclusions

The current study has shown that IPS accurately predicts LS. Using the DT machine learning procedure, we showed that IPS indicators were able to discriminate between cows with different locomotion scores. While using specificity/sensitivity analysis, we found that using a threshold of at least two positive indicators, IPS had a high specificity and sensitivity for detecting clinically lame cows (locomotion scores ≥ 2 on a scale of 0 to 3). Thus, our results suggest that the IPS technique has significant potential to be used as an alternative for detecting lameness in pasture-based dairy herds. However, this was a small study on a convenience sample of only two farms, so further research is required before IPS could replace LS. This investigation should focus on:

(1) Establishing the relationship between IPS and LS across more farms with different milking parlours and different prevalence of lameness and across more observers.
(2) Identifying whether the IPS procedure can be improved further to address issues with time for scoring (increasing the proportion of cows that can be scored per milking) and visibility of indicators.
(3) Determining whether IPS can reliably differentiate cows with locomotion score 1, which should only be monitored, from cows with locomotion score ≥2, which need examination and treatment.

## Figures and Tables

**Table 1:** The description of locomotion scoring according to the DairyNZ system [39].

Score	Clinical Term	Evaluation Criteria
0	Sound	The cow walks confidently, even weight-bearing and tracks up.
1	Imperfect locomotion	The cow walks unevenly, does not track up, with a mildly arched back when walking.
2	Lame	An arched back, the favoured limb moves faster than the lame leg, feet placed unevenly, head bobs up and down when walking.
3	Severely lame	They walk very slow, reluctant to bear weight, arched back, and head bobs obvious.

**Table 2:** The checklist of indicators that were put forward for use during the in-parlour scoring procedure (identified from the literature).

Indicator	Description
Shifting weight (SW)	Frequent changing of feet, i.e., twice or more per 30 s
Abnormal weight distribution (AWD)	The asymmetric placing of the claws on the ground
Swollen heel or hock joint (SHH)	Abnormal swelling of the heel and surrounding tissues (observed from the plantar aspect of the foot) or hock joint
Overgrown hoof (OH)	Irregular growth of claw capsule on at least one hind limb
Observed claw injury (OCI)	Observation of claw injury of any type, i.e., bruises, cuts
Swelling/separation around the coronary band (SCB)	Abnormal swelling or separation around the coronary band
Arched back (AB)	Arching of the back while standing

**Table 3:** Locomotion score of cows with the in-parlour scoring results available for both farms. The number of observations per farm was 3006 on farm 1 and 1119 on farm 2.

Month	Farm 1: LS				Farm 2: LS			
	LS 0	LS 1	LS 2	LS 3	LS 0	LS 1	LS 2	LS 3
August	220	82	8	0	73	31	4	0
September	226	95	5	0	70	43	5	0
October	228	101	8	1	67	50	3	1
November	240	85	10	1	89	32	5	0
December	254	86	10	0	93	24	5	0
January	246	81	12	2	87	36	5	1
February	260	63	9	0	80	39	4	1
March	261	66	9	0	89	42	3	0
April	197	122	15	3	93	41	3	0
**Total**	**2132**	**781**	**86**	**7**	**741**	**338**	**37**	**3**
**% of the total by farm**	**70.9**	**26.0**	**2.9**	**0.2**	**66.2**	**30.2**	**3.3**	**0.3**

LS = locomotion score; total denotes the sum of locomotion scores.

**Table 4:** Distribution of in-parlour scoring indicators with a locomotion score available. The number of observations per farm was 3006 on farm 1 and 1119 on farm 2.

Month	In-Parlour Scoring Results—Farm 1											
	SW0	SW1	AWD0	AWD1	SHH0	SHH1	OH0	OH1	Total0	Total1	Total2	Total3
August	284	26	280	30	294	16	274	36	222	72	14	2
September	297	29	286	40	312	14	287	39	215	102	7	2
October	311	27	312	26	308	30	297	41	230	92	16	0
November	318	18	314	22	314	22	281	55	235	86	14	1
December	320	30	324	26	339	11	303	47	251	86	12	1
January	315	26	315	26	323	18	297	44	243	82	16	0
February	317	15	315	17	323	9	287	45	256	67	8	1
March	311	25	313	23	328	8	296	40	251	75	9	1
April	299	38	290	47	320	17	268	69	191	124	19	3
**Total**	**2772**	**234**	**2749**	**257**	**2861**	**145**	**2590**	**416**	**2094**	**786**	**115**	**11**
**% of total**	**92.2**	**7.8**	**91.4**	**8.6**	**95.2**	**4.8**	**86.2**	**13.8**	**69.6**	**26.2**	**3.8**	**0.4**
	**In-parlour scoring results—farm 2**											
August	102	6	100	8	100	8	88	20	70	34	4	0
September	114	4	106	12	108	10	80	38	61	51	5	1
October	111	10	111	10	112	9	86	35	64	52	3	2
November	121	5	121	5	114	12	104	22	88	33	4	1
December	117	5	116	6	114	8	104	18	91	26	4	1
January	118	11	117	12	124	5	107	22	87	35	6	1
February	116	8	108	16	117	7	97	27	76	38	10	0
March	124	10	122	12	128	6	108	26	88	39	6	1
April	129	8	123	14	131	6	108	29	88	43	4	2
**Total**	**1052**	**67**	**1024**	**95**	**1048**	**71**	**882**	**237**	**713**	**351**	**46**	**9**
**% of total**	**94.0**	**6.0**	**91.5**	**8.5**	**93.7**	**6.3**	**78.8**	**21.2**	**63.7**	**31.4**	**4.1**	**0.8**

SW0 = weight shifting absent, SW1 = weight shifting present, AWD0 = abnormal weight distribution absent, AWD1 = abnormal weight distribution present, SHH0 = swollen heel or hock joint absent, SHH1 = swollen heel or hock joint present, OH0 = overgrown hoof absent, OH1 = overgrown hoof present. Total means number of positive indicators observed. Total0 = no positive indicator observed, Total1 = only one positive indicator observed, Total2 = two positive indicators observed, Total3 = three positive indicators observed. No cow on either farm was observed with four positive indicators.

**Table 5:** Phi coefficients for association between presence/absence of in-parlour scoring (IPS) indicators, and presence/absence of other in-parlour scoring indicators, and locomotion scores ≥ 2. (4125 paired observations across two farms; *n* = 3006 on-farm 1 and 1119 on-farm 2).

IPS Indicators	LS	SW	AWD	SHH	OH
SW	0.405		−0.029	0.127	−0.053
AWD	0.429			0.103	−0.040
SHH	0.490				0.041
OH	0.572				

LS = locomotion score, SW = shifting weight, AWD = abnormal weight distribution, SHH = swollen heel or hock joint, OH = overgrown hoof.

**Table 6:** Sensitivity and specificity with 95% confidence interval for detecting locomotion score ≥2. Data amalgamated across farms (*n* = 4125).

IPS Indicators Present	Sensitivity	Specificity	PPV	NPV
SW	42.1 (33.6–51.0)	93.9 (93.1–94.6)	18.6 (5.3–22.4)	98.0 (97.7–98.3)
AWD	47.4 (38.7–56.2)	92.8 (91.9–93.6)	17.9 (15.0–21.2)	98.1 (97.8–98.4)
SHH	76.7 (68.6–83.6)	97.1 (96.6–97.6)	47.2 (42.2–52.3)	99.2 (98.9–99.4)
OH	42.9 (34.3–51.7)	85.1 (83.9–86.2)	8.7 (7.2–10.6)	97.8 (97.5–98.1)
One or more (≥1)	100.0 (97.3–100.0)	70.3 (68.8–71.7)	10.1 (9.7–10.5)	100.0
Two or more (≥2)	93.2 (87.5–96.9)	98.6 (98.2–98.9)	68.5 (62.6–73.9)	99.8 (99.6–99.9)
Three (3)	15.0 (9.4–22.3)	100.0 (99.9–100.0)	100.0	97.3 (97.1–97.4)

SW = shifting weight, AWD = abnormal weight distribution, SHH = swollen heel or hock joint, OH = overgrown hoof, PPV = positive predictive value, NPV = negative predictive value. Note that results for each farm separately are presented in Appendix A Table A1.

**Table 7:** Confusion matrix of the decision tree classifier with the highest test accuracy calculated based on a test set including 1030 observations.

	Classified by Optimal Decision Tree Classifier		
Locomotion Score	Sound	Imperfect Gait	Lame or Severely Lame
Sound (0)	697	21	0
Imperfect gait (1)	4	274	2
Lame or severely lame (≥2)	0	8	24

**Table 8:** True positive and false-positive rates and precision of the decision tree classifier with the highest test accuracy calculated based on a test set including 1030 observations.

Locomotion Score	True Positive Rate	False Positive Rate	Precision
0	97.1%	1.3%	99.4%
1	97.9%	3.9%	90.4%
≥2	75%	0.2%	92.3%

## Data Availability

Data are available at request from the corresponding author.

## Appendix A

**Table A1:** Sensitivity and specificity with 95% confidence interval for detecting lame cows (locomotion score ≥2) the number of observations on farm 1 (*n* = 3006), and on farm 2 (*n* = 1119).

IPS Indicators Present	Sensitivity	Specificity	PPV	NPV
SW	46.2 (35.8–56.9)	93.4 (92.5–94.3)	18.4 (14.8–22.6)	98.2 (97.8–98.5)
AWD	44.1 (33.8–54.8)	92.6 (91.6–93.5)	16.0 (12.7–19.8)	98.1 (97.7–98.4)
SHH	76.3 (66.4–84.5)	97.5 (96.8–98.0)	49.0 (42.7–55.2)	99.2 (98.9–99.5)
OH	38.7 (28.8–49.4)	87.0 (85.7–88.2)	8.7 (6.7–11.1)	97.8 (97.4–98.1)
One or more (≥1)	100.0 (96.1–100.0)	71.8 (70.1–73.4)	10.2 (9.7–10.7)	100.0
Two or more (≥2)	92.5 (85.1–96.9)	98.6 (98.1–99.0)	68.3 (61.1–74.6)	99.8 (99.5–99.9)
Three (3)	11.8 (6.1–20.2)	100.0 (99.9–100.0)	100.0	97.3 (97.1–97.5)
SW	32.5 (18.6–49.1)	95.0 (93.5–96.2)	19.4 (12.6–28.8)	97.4 (96.8–97.9)
AWD	55.0 (38.5–70.7)	93.2 (91.6–94.7)	23.2 (17.4–30.1)	98.2 (97.5–98.7)
SHH	77.5 (61.6–89.2)	96.3 (95.0–97.3)	43.7 (35.4–52.3)	99.1 (98.5–99.5)
OH	52.5 (36.1–68.5)	80.0 (77.5–82.3)	8.9 (6.6–11.8)	97.9 (97.0–98.4)
One or more (≥1)	100.0 (91.2–100.0)	66.1 (63.2–68.9)	9.9 (9.1–10.6)	100.0
Two or more (≥2)	95.0 (83.1–99.4)	98.4 (97.5–99.1)	69.1 (58.1–78.3)	99.8 (99.3–99.9)
Three (3)	22.5 (10.8–38.5)	100.0 (99.7–100.0)	100.0	97.2 (96.7–97.6)

SW = shifting weight, AWD = abnormal weight distribution, SHH = swollen heel or hock joint, OH = overgrown hoof, LS = locomotion score, PPV = positive predictive value, NPV = negative predictive value, Total-denotes the number of observed indicators.
